# Challenges in translational machine learning

**DOI:** 10.1007/s00439-022-02439-8

**Published:** 2022-03-04

**Authors:** Artuur Couckuyt, Ruth Seurinck, Annelies Emmaneel, Katrien Quintelier, David Novak, Sofie Van Gassen, Yvan Saeys

**Affiliations:** 1 Department of Applied Mathematics, Computer Science and Statistics, Ghent University, Gent, Belgium; 2 Data Mining and Modeling for Biomedicine, VIB-UGent Center for Inflammation Research, Gent, Belgium; 3 Department of Pulmonary Diseases, Erasmus MC, Rotterdam, The Netherlands

## Abstract

Machine learning (ML) algorithms are increasingly being used to help implement clinical decision support systems. In this new field, we define as “translational machine learning”, joint efforts and strong communication between data scientists and clinicians help to span the gap between ML and its adoption in the clinic. These collaborations also improve interpretability and trust in translational ML methods and ultimately aim to result in generalizable and reproducible models. To help clinicians and bioinformaticians refine their translational ML pipelines, we review the steps from model building to the use of ML in the clinic. We discuss experimental setup, computational analysis, interpretability and reproducibility, and emphasize the challenges involved. We highly advise collaboration and data sharing between consortia and institutes to build multi-centric cohorts that facilitate ML methodologies that generalize across centers. In the end, we hope that this review provides a way to streamline translational ML and helps to tackle the challenges that come with it.

## Introduction

Advances in biomedicine go hand in hand with the rise of frontier technologies that often generate complex and high-dimensional data. To unlock the full potential of these data, novel advances in machine learning (ML) are finding their way to the clinic. ML, a sub-field of the broader domain of artificial intelligence (AI), is an umbrella term for algorithms that learn a model directly from data. It is a highly interdisciplinary and still-evolving field with contributions from computer science, mathematics and statistics, that is currently at the forefront of life sciences. We will refer to the use of ML in a clinical environment as “translational machine learning”. It focuses on any use of ML as a decision support system in the clinic, where the algorithm provides additional information that can help the clinician to better treat the patient (Moreau et al. 1997; Rubio et al. 2010).

Many clinical applications of ML include image-based technologies (e.g., MRI scans, skin pictures for dermatology, etc.) where deep learning (DL) methods have often outperformed clinicians (Esteva et al. 2017; Watson et al. 2019; Aggarwal et al. 2021). ML is also established in other fields such as epigenomics (Corces et al. 2020) and genomics (Shipp et al. 2002; Ye et al. 2003; Tabl et al. 2019). Recently, similar techniques have also been explored in the area of high-throughput, single-cell technologies, such as single-cell RNA-sequencing (Tang et al. 2009), (spectral) flow cytometry (Fulwyler 1965; Nolan and Condello 2013) and mass cytometry (Bandura et al. 2009). Due to the high-dimensional nature of these data, with up to millions of cells (data points) and tens to thousands of genes, proteins or other biological features measured (dimensions), it becomes infeasible to extract relevant information without computational techniques. In this review, we will focus on translational applications of ML in the single-cell field and add examples from other relevant fields.

### Machine learning overview

**Fig. 1:**
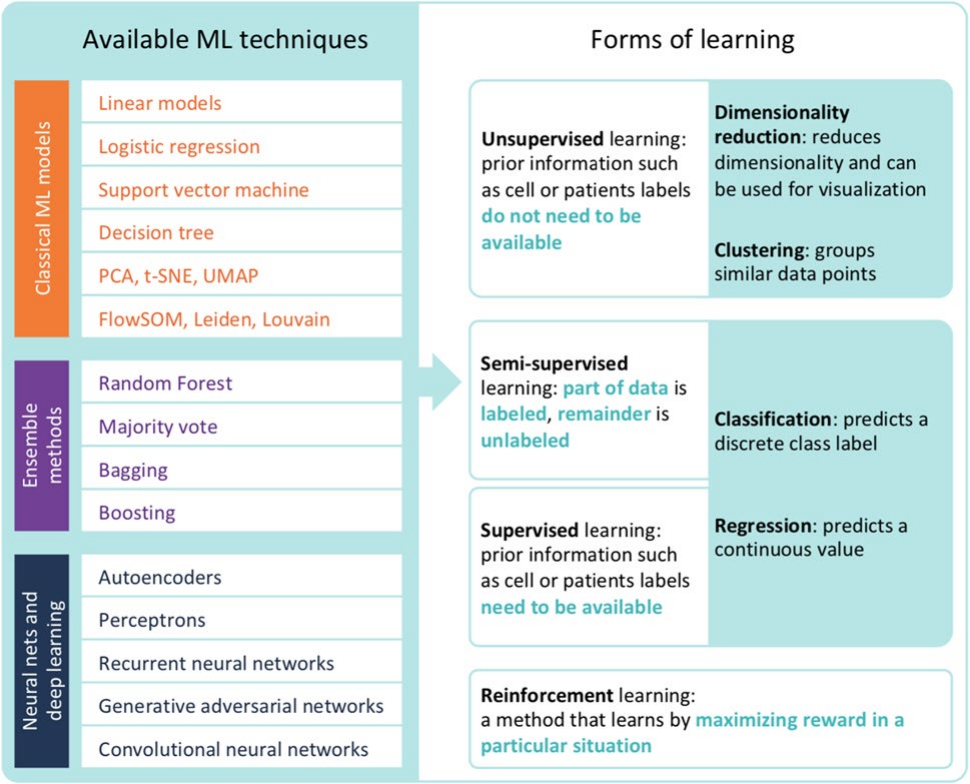
Overview of machine learning techniques and the forms of learning ML is capable of doing. *ML* machine learning, *PCA* principal component analysis, *t-SNE* t-distributed neighbor embedding, *UMAP* uniform manifold approximation and projection

ML algorithms can be organized by (a) the underlying techniques and (b) the type of learning they use to model the data (Fig. 1). Four different ways of how the model learns from the data can be distinguished: unsupervised learning, supervised learning, semi-supervised learning and reinforcement learning. Below, we give a short overview of how various ML algorithms can be applied to several steps in translational research.

### Unsupervised learning

In data exploration, when no prior information or outcome such as patient treatment or survival is used, one can apply unsupervised ML to find previously unknown data patterns, e.g., to perform patient stratification. Unsupervised ML can be further subdivided into dimensionality reduction and clustering analysis. Dimensionality reduction is used to decrease data complexity and allows, for example, to visualize information from many parameters in a two-dimensional plot. Clustering is used to find groups of similar data points, both on a patient level and on a cell level. During data exploration, outliers and technical variation, such as batch effects or other technical artifacts, are typically also identified. This can be done with unsupervised ML or with data cleaning tools.

Principal component analysis (PCA) is the best-known linear dimensionality reduction method, where as much as possible of the data’s variation is preserved. It was, among others, applied to improve the Catovsky–Matutes Score that distinguishes Chronic Lymphocytic Leukemia (CLL) from non-CLL (Moreau et al. 1997; Jalal 2021) and to discriminate mild versus moderate Alzheimer’s disease (Pagani et al. 2009). Widespread examples of non-linear dimensionality reduction algorithms are t-distributed Stochastic Neighbor Embedding (t-SNE) (van der Maaten and Hinton 2008) and Uniform Manifold Approximation and Projection (UMAP) (McInnes et al. 2018), which project high-dimensional data on a latent space while preserving local and/or global structure. Both are mainly used for graphical representation. Although sample labels are not used by the technique itself, they can still be used to visually discover, e.g., treatment or disease-specific populations. In a study by Esteva et al. (2017), t-SNE was used to visualize a hidden layer of a deep neural network that was used to classify skin cancer, and in Zou et al. (2020), UMAP was used to visualize ACE2 expression in SARS-CoV-2 infection.

Examples of clustering algorithms often used for single-cell data are, among others, community-detection-based algorithms such as Louvain (Blondel et al. 2008) and Leiden clustering (Traag et al. 2019) and methods based on self-organizing maps, such as FlowSOM (Van Gassen et al. 2015), a standard method in the cytometry field. Leiden clustering, mentioned in Zhang et al. (2019), was used to explore the immune landscape of single-cell RNA-seq data of hepatocellular carcinoma, and in Gaebler et al. (2021), FlowSOM clustering was performed to track the evolution of immunity in COVID-19.

### Supervised learning

Once data exploration is done, the following step is typically a more supervised approach. Supervised ML is a form of ML where prior external information, such as the health status of a patient, is available. This external information can be used to build a model (often referred to as “training” the model) which, in turn, is able to predict the status of new, unseen samples. In supervised ML, we mainly distinguish between classification, which predicts a discrete class label, and regression, which predicts a continuously valued quantity. In Chiofolo et al. (2019), a random forest classification model was used to classify high-risk patients with regard to acute kidney injury, and Akyea et al. (2020) compared five supervised classification ML techniques to predict hypercholesterolemia, both using clinical patient data. In Smith et al. (2013), the researchers compared random forest regression and multiple linear regression to predict concentrations of a neurochemical based on the concentrations of other neurochemicals, and in Seiler et al. (2021), the authors applied multiple regression to assess differences in response to IFN-α stimulation in early and late pregnancy using mass cytometry data.

### Semi-supervised learning

The combination of supervised and unsupervised learning, where a part of the data is labeled and the remainder is unlabeled, is called semi-supervised learning. This is especially useful in those cases where labeling samples is expensive or difficult. It has the potential to improve accuracy compared to only using the labeled data in supervised learning thanks to the additional data, which might clarify underlying data structures that are not as strongly pronounced in the limited labeled dataset. In contrast, labeled data can also improve unsupervised learning thanks to the inclusion of prior information (Zhu and Goldberg 2009). Semi-supervised learning works by combining both labeled and unlabeled data points to improve the model, compared to using only either labeled or unlabeled data. Zhai et al. (2020) proposed semi-supervised learning in combination with a convolutional neural network (CNN) to detect supraventricular ectopic beats in electrocardiograms, and Shi and Zhang (2011) tested low-density separation, a semi-supervised learning technique, on a colorectal cancer dataset to detect recurrence.

### Reinforcement learning

Another form of ML, next to supervised, unsupervised and semi-supervised learning, is reinforcement learning. This method learns to take an optimal sequence of actions to maximize the cumulative reward which is a measure of how good a certain goal was achieved. For example, an effective treatment strategy for sepsis with certain treatment doses or frequencies was learned based on patient mortality as a reward (Komorowski et al. 2018). It was also used to extract patient-specific treatment strategies against cancer from only clinical data (Zhao et al. 2009). Liu et al. (2020a) surveyed the literature on the use of reinforcement learning in clinical decision support and its challenges.

### Deep learning

Deep learning (DL), a subcategory of ML that can be applied in (semi)-supervised, unsupervised and reinforcement learning, is currently considered state-of-the-art in many classification problems (Esteva et al. 2019; Topol 2019). It is based on artificial neural networks which are inspired by the human brain. One particular aspect of DL is that it can learn feature representations (representation learning) which sets it apart from classical ML techniques that learn from a given set of features. Initially, this was mostly used for image-related tasks, where relevant features can automatically be extracted from the pixel values, avoiding the need for an upfront definition of features of interest and manual annotation of the images. This was for example used to extract more prognostic information from tissue slides of colorectal cancer (Bychkov et al. 2018). More recently, DL-based methods have also been used in many other bioinformatics tasks, e.g., involving different types of omics data. For example, Eraslan et al. (2019) described the use of DL in genomic applications. scGNN, as mentioned in Wang et al. (2021), is a graph neural network developed for single-cell RNA-seq and model cell–cell interactions, and in Arvaniti and Claassen (2017), a CNN was used to detect rare disease-specific cells from cytometry data. A disadvantage is that deep learning is prone to overfitting when applied to small sample sizes. Methods such as data augmentation, transfer learning, and self-supervised learning can circumvent this problem (Lu et al. 2006; Mieth et al. 2019; Marouf et al. 2020).

Most of the time, these DL algorithms are known to be “black boxes” and are not intuitive or interpretable. A lot of research currently goes into making these models more interpretable without making them less accurate (Ahmad et al. 2018). A new branch in AI that currently tries to tackle these “black box” issues is explainable AI (XAI) (Gunning et al. 2019).

### Translational machine learning

**Fig. 2:**
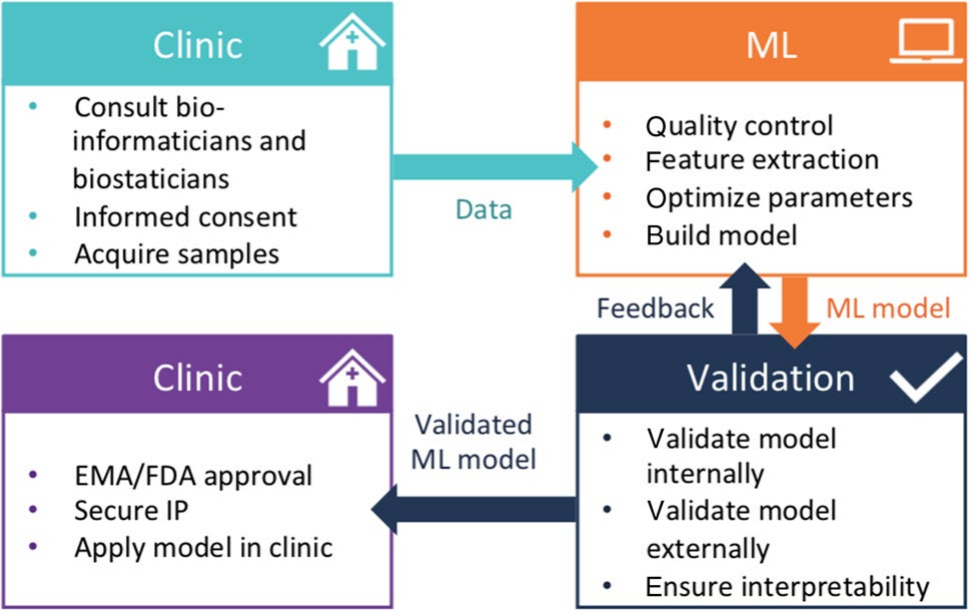
The process of translational machine learning. *EMA* European Medicines Agency, *FDA* Food and Drug Administration, *IP* intellectual property, *ML* machine learning

In translational machine learning, an ML model is used in the clinic as a decision support system. Before actually adopting these systems in the clinic, several steps need to be completed. First of all, the model needs to be externally validated. Next, the intellectual property needs to be secured, and approval is needed from the respective authorities, e.g., from the U.S. Food and Drug Administration (FDA) or the European Medicines Agency (EMA) (Fig. 2). Applications of translational ML are widespread across many clinical disciplines, such as oncology, endocrinology and radiology. A review by Benjamens et al. (2020) surveyed the literature for AI- or ML-assisted devices and algorithms that were approved by the FDA as “software as a medical device” and came up with 29 examples. Two recent examples the authors mention are the Eko analysis software as a deep learning algorithm for cardiac murmur using a digital stethoscope platform (Chorba et al. 2021) and QuantX, which improves diagnosis of breast cancer based on MRI scans (Benjamens et al. 2020; Jiang et al. 2021).

To our knowledge, up to now, no translational ML models for single-cell data have been approved by the FDA. Nevertheless, ML algorithms are already being used in the clinic to analyze single-cell data. One example is Infinicyt, a widely used software to analyze flow cytometry data which uses automatic clustering based on an in-house database to make analysis faster and easier for the clinicians (Pedreira et al. 2019).

**Fig. 3:**
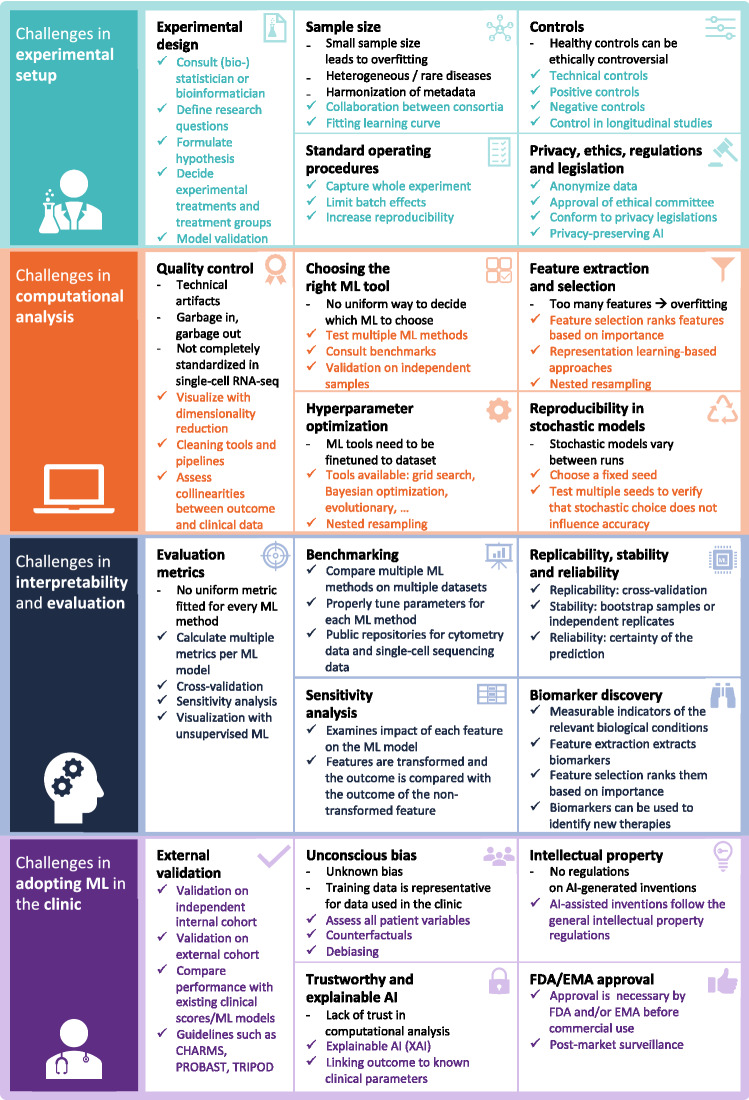
Overview of challenges in translational ML. *AI* artificial intelligence, *EMA* European Medicines Agency, *FDA* Food and Drug Administration, *ML* machine learning, *XAI* explainable AI

In this review paper, we will discuss the challenges that emerge in translational ML (Fig. 3). We will go through the whole process of a clinical application, including experimental setup, computational analysis, interpretation of results, evaluation of ML methods and application in the clinic. We will highlight the challenges involved and propose solutions to overcome these.

## Challenges in experimental setup

The first step in translational ML is the experimental setup. The experimental design and choice of an appropriate sample size are important aspects to consider, together with selecting controls and creating standard operating procedures (SOPs). Furthermore, potential sources of technical variation such as the date of acquisition, need to be identified. SPIRIT-AI (Cruz Rivera et al. 2020), CONSORT-AI (Liu et al. 2020b) and MI-CLAIM (Norgeot et al. 2020) are guidelines concerning the use of AI in clinical trials and state which aspects need to be considered to improve transparency, interpretability and reportability before starting. They recommend, for example, to specify the intended use of the AI model or to state the exclusion or inclusion criteria at the level of the input data or at the level of participants.

### Experimental design

Consulting with a (bio-)statistician and bioinformatician prior to the experimental setup is strongly advised. Just as in regular studies, ML experiments in general require an optimized experimental design to ensure that the biological signal of interest can be disentangled from confounding technical and biological sources of variation. Defining the research questions and variables to measure is the first step in experimental design. After an in-depth literature study, gaps in the current knowledge can be identified, and research questions of interest can be defined. From these research questions, hypotheses are distilled, and the experimental design is decided. This takes into account, among others, which samples for model building and validation are available at the time of clinical decision-making. Experimental design also determines patient inclusion and exclusion criteria, endpoints, dose of the treatments, relevant control groups and distribution of the patients over the treatment groups. This last step can be done in a completely (stratified) randomized design or a (stratified) randomized block design (Lundstedt et al. 1998; Kreutz and Timmer 2009). Randomized control trials are often recommended, because they are less sensitive to selection bias, avoiding a specific subset of patients getting a specific treatment, which would confound the treatment effect with the underlying patient condition. On the other hand, observational studies can yield complementary information, as they can be used to assess how well the model can be used to predict unseen data in a more representative and heterogeneous sample of the clinical population, using less strict inclusion criteria (Hannan 2008).

During predictive modeling, validation of the model is a crucial aspect of supervised ML studies that should already be included in the design of the study. A part of the cohort will be used as training data to develop the model, and some samples will be withheld to test the performance of the model. This type of validation is also known as internal validation. External validation, in contrast, refers to testing model performance on a completely independent and external cohort. Both types are important when validating an ML model.

Besides splitting data from a cohort into two parts—a training and a test dataset—the data can be split in a set of *k*-fold for cross-validation. The model is trained on *k* – 1-fold and tested on the remaining fold. The procedure is repeated, iterating over all the folds, and the outcome is a summary measure for the performance across all folds (Browne 2000). Optimization of hyperparameters and optimal feature subset selection require more complex designs with nested cross-validation, to avoid overestimating the performance of the model (Vabalas et al. 2019).

### Sample size

A sufficient number of samples are required to ensure generalizable ML results which do not overfit the training data used to develop the ML model. This was demonstrated in a review on neuroimaging data by Varoquaux (2018), where the authors show that a small sample size results in a larger error variance which leads to overfitting and confirmation bias. An overfitted ML model is typically too complex and does not generalize well to previously unseen data.

The appropriate minimum number of patients or samples in a training set can be estimated by fitting a learning curve to the relationship between training set size and a measure of classifier performance such as, e.g., accuracy or sensitivity (Figueroa et al. 2012). First, a minimum required performance level is determined. This measure depends on the specific study, e.g., if there is class imbalance, or if sensitivity is more important than specificity. Next, the performance is evaluated with an initial minimal subset of training samples. The process is repeated, each time with a larger subset, until all available training samples are used. Finally, a weighted learning curve is fit to infer the minimum training set size associated with the required performance. The challenge remains to start a pilot study with a sample size that is large enough to fit a learning curve.

The recent MultiML algorithm expands this approach for multi-omic datasets, evaluating the relative contribution of different omics types (Tarazona et al. 2020). This allows researchers to assess how many patients and omics types to include to achieve the required classifier performance.

Statistically relevant numbers often turn out infeasible for rare diseases with, e.g., a prevalence of fewer than 5 patients per 10,000 (European Commission 2021). A study by Schaefer et al. (2020) reviewed 211 studies on 74 different rare diseases and showed that diseases with a higher prevalence were investigated more frequently than diseases with a lower prevalence. Only a small proportion of these studies confirmed their ML models on an external dataset (Schaefer et al. 2020). The same study mentioned that ML was mostly used for diagnosis or prognosis, while studies to improve treatment were uncommon (Schaefer et al. 2020).

Another challenge that correlates with increasing sample sizes is the harmonization of clinical metadata; for example, patient labels might differ between institutions in spelling or in the way they are defined. A possible solution for both increasing sample sizes and aligning metadata could be stronger collaborations between research institutes and the establishment of worldwide consortia. This would not only increase sample sizes but also lead to better standardization of equipment between the institutes, resulting in larger, more harmonized datasets and more widely applicable ML models. Larger sample sizes would also benefit research into heterogeneous diseases, such as acute myeloid leukemia (Li et al. 2016; van Galen et al. 2019). On one hand, large variability between patients suffering from the same disease decreases the statistical power, e.g., for differential diagnosis, and a larger sample size will be required to counteract this. On the other hand, including a wider variety of patients will ensure the broader applicability of the ML algorithm.

### Controls

To guarantee data quality, it is important to take along controls during the experiments. This can include positive and negative controls. A positive control validates how strong a true-positive signal can be, and a negative control how much background noise is to be expected from a negative signal.

Biologically, it is important to take the relevant control populations into account, depending on the clinical research question. For instance, when investigating a disease, it might be relevant to also measure the status of (age and/or gender-matched) healthy donors or patients with a different disease with similar symptoms (diseased controls). When investigating a treatment, a patient group treated with a placebo, or the standard-of-care treatment should also be taken along as negative control. In some cases, it might be unethical to use samples that require an invasive procedure, such as surgery of a healthy person. In those cases, it is sometimes possible to recruit patients with another well-defined medical issue that would require a similar operation as a control group.

Single-cell technologies might require additional technical controls. In a cytometry setting, single stains of beads or cells are used as positive controls, while fluorescence-minus-one stainings (FMOs) are used to estimate the negative background distribution. Equivalent controls in transcriptomics are technically much more challenging and expensive. One possibility is the addition of cross-species spike-in cells (Marquina-Sanchez et al. 2020).

Next to controls for signal strength, controls for signal stability can be taken along. This is crucial if the experiment takes place over a longer period, on multiple machines and/or between multiple laboratories. In those cases, it is recommended to aliquot a sample that can be taken along in each batch as a technical replicate. Researchers should ensure that this technical control expresses all relevant features of the real samples under investigation. Therefore, it might be necessary to use a combination of multiple samples. Having sufficient biological and technical controls will allow a baseline to be established and will result in a more accurate interpretation of the experimental outcomes.

### Standard operating procedures

SOPs are vital for reproducibility and reducing technical variation. This is not only important for the experiment itself, but also for comparisons with future experiments or across different institutes. SOPs capture everything from the reagents, scoring and reporting methods, sample collection, handling and storage, to guidelines for data generation, consistent data analysis, file formats and so on (McShane et al. 2013; Rybakowska et al. 2020). All this helps to reduce the technical variation or batch effects to a minimum when the experiment is repeated in the future or by different research groups. In flow cytometry, batch effects can be limited by calibrating the machine with beads with a known fluorescence and size (Wang and Hoffman 2017), while in single-cell sequencing, cell hashing (a technique where multiple samples are loaded in the same well, Stoeckius et al. 2018) can be used to reduce batch effects.

### Privacy, ethics, regulations, and legislation

A final important element of the experimental setup is compliance with ethics, regulations and laws. Informed consent is required of each patient for using their data in research, and the ethics committee has to approve the experiment. Moreover, it is advised to keep patient organizations in the loop throughout the whole process, so that they are up-to-date, get a broad overview of the methodology and understand the potential benefits from a patient viewpoint. This will also help to build trust and stimulate the adoption of ML techniques in the clinic.

Before the data are shared with bioinformaticians or data scientists, the clinician also needs to anonymize or pseudonymize the data, to ensure the patient's privacy and to conform to regulations such as the General Data Protection Regulation (GDPR). Nevertheless, research by Rocher et al. (2019) re-identified up to 99.98% of Americans using their Gaussian copula-based method based on 15 demographic attributes, which proves that pseudonymization of datasets is not as trivial as it might seem. Similarly, Schwarz et al. (2019) showed that face-recognition software could identify individuals from reconstructed cranial MRI scans. Another approach is using privacy-preserving ML methods. These provide a way to include ML in the pipeline without violating the privacy of the patients and to build collaborative models across institutes, even without the need to exchange sensitive patient data (Beaulieu-Jones et al. 2019; Kaissis et al. 2020).

Once the experimental design is set up, the sample size and demographic space are established, the SOPs are defined, and everything is approved by the ethics committee, the data can be acquired, and the downstream computational analysis can be initialized. Before doing the real experiment on the anticipated cohort, it makes sense to already test and optimize all techniques and analyses during a pilot experiment.

## Challenges in computational analysis

### Quality control

One of the first things to do when starting the computational analysis is to assess the quality of the data and to clean the data if technical artifacts are detected. As the saying “garbage in, garbage out” illustrates, low-quality data will lead to low-quality results. Therefore, ensuring high-quality data is a key step in computational analysis. Luckily, many cleaning tools and pipelines exist. Assessing the data quality can be done in several ways. Dimensionality reduction, an unsupervised ML technique, can be used as an exploration tool. A lower dimensional embedding is produced to give a first impression of the structure of a given dataset. Additionally, coloring individual points by sample ID or other technical variables recorded can reveal possible batch effects (Nowicka et al. 2019). Another aspect to acknowledge is if there are any colinearities between the desired outcome and the cohort clinical data, such as age or gender. Assessing and including these in the downstream analysis are important to reduce bias and to delineate the confounder from the outcome.

Single-cell RNA-seq quality can be evaluated with a range of tools throughout the pipeline. It is common to first distinguish empty droplets from cells and then filter on gene counts and the relative amount of mitochondrial RNA (Luecken and Theis 2019). These filter settings are not standardized and often require iterative adjustments to optimize the quality for each specific sample (Stegle et al. 2015; Lun et al. 2016). In a later stage of the analysis, more advanced algorithms can be used to identify doublets in single-cell RNA-seq data (Xi and Li 2021) or to correct for batch effects when integrating multiple samples (Chazarra-Gil et al. 2021).

While in cytometry data, quality control is still often done manually, new computational tools such as PeacoQC (Emmaneel et al. 2021) or flowAI (Monaco et al. 2016) have also been developed to identify and potentially remove outliers.

### Choosing the right ML model

Choosing the right ML model for your analysis is a crucial step and strongly depends on the research question. There is no uniform way to decide a priori which ML algorithm is compatible with your data, and therefore, comparing multiple algorithms on the data at hand or consulting algorithm benchmarks is recommended (Weber and Robinson 2016; Saelens et al. 2019; Chazarra-Gil et al. 2021). The use of autoencoders, for example, is state-of-the-art in DL methods for processing images (Uzunova et al. 2019) and single-cell RNA-seq data (Grønbech et al. 2020). In cytometry, the use of autoencoders is also on the rise, with uses in dimensionality reduction (Szubert et al. 2019) and differential analysis (Arvaniti and Claassen 2017), but not yet widely adopted.

The specific hierarchical structure of single-cell data, where features provide information at the cell level but the outcome of interest (e.g., diagnosis) is at the patient level, is a case of learning (Herrera et al. 2016). This implies an additional step in the ML model to infer the patient label from the thousands of cells (or instances) of that patient (or bag). There are three main approaches to resolve this issue. In mapping-based approaches, the information on the cells is summarized per patient in, for example, cell type percentages, and these are used as input for the ML model. Instance-based approaches classify the individual cells as, e.g., diseased and use a decision rule to infer the patient labels from the cell labels. Finally, bag-based approaches use distance functions that capture the similarity between patients as input for modified ML models. Weber et al. (2019) and Nowicka et al. (2019) proposed mapping-based approaches on single-cell data, whereas Cheplygina et al. (2014) and Xiong et al. (2021) compared both instance-based and bag-models on imaging data and next-generation sequencing data, respectively.

Before starting the computational pipeline, the data need to be prepared for internal validation, as explained earlier. The data can be split into a training and a test set, to train and validate the model, respectively. It needs to be emphasized that the test set can only be used to validate the model and none of its information can be used while building the ML model as this would introduce data leakage and would eventually lead to an incorrect estimation of the performance of the ML model. For more complex settings, e.g., for parameter optimization or feature selection, nested resampling methods can be used. The final assessment of the performance is achieved when validating the ML model on an external dataset (external validation), e.g., from other institutes, and this is a crucial step when aiming for broadly applicable models.

### Feature extraction and selection

The next step after choosing the right ML model is feature extraction and feature selection. Features can be extracted from the original high-dimensional space, for example (ratios or sums of) cell type abundances, quantification of gene or protein expression and so on. Importantly, the features need to be available and measurable at the time of prediction. If cell types are not available, cluster labels can be used instead. Features can also be extracted from a latent space after dimensionality reduction. Generally, features are abundant which is why feature selection is important. Feature selection and, as previously mentioned, an acceptable number of samples (Varoquaux 2018) reduce overfitting and training time and increase accuracy (Saeys et al. 2007). Reducing overfitting is important, since we do not want the model to fit on noise instead of real biological patterns. Some modeling approaches, such as Lasso regression (Tibshirani 1996) or elastic net regression (Zou and Hastie 2005), implicitly perform feature selection and do not require any a priori feature selection and might therefore be easier to implement. Similarly, representation learning-based approaches take care of feature engineering themselves. Since there is not one optimal feature selection technique, it is recommended to try multiple.

We want to highlight the use of feature selection in combination with resampling procedures where feature selection is only performed on the training dataset. In combination with nested resampling, the feature selection is performed on each iteration of the inner resampling. Nevertheless, it needs to be noted that features can change from fold to fold. For interpretability, typically, a final model is trained using all data (i.e., without any resampling), in which the same procedure for feature selection (and/or parameter optimization) is applied. Another challenge arises when using feature selection in combination with multiple sources of high-dimensional data with largely varying numbers of features, as it needs to be avoided that one source dominates the other (Baldwin et al. 2020).

We also want to emphasize the relative importance of demographic and clinical features such as age, comorbidities, etc., compared to features from high-throughput data. Volkmann et al. (2019) found that adding features from omics data only caused a small increase in the predictive value of a model if the model already contained a substantial amount of clinical features. This means that clinical data can already hold valuable information when used in ML models, and it should always be verified that the omics data gives relevant additional insights.

### Hyperparameter optimization

ML tools do not come as “one-size-fits-all” models, as they rely on hyperparameters that need optimizing for every dataset to maximize performance. Hyperparameters used to tune the learning process by the end-user (e.g., certain thresholds to be specified) differ from parameters that are learned by the ML algorithm itself (e.g., a node weight in the case of neural networks), and these often have to be fine-tuned to maximize predictive performance. Many tools have been developed for hyperparameter optimization. Grid search employs an exhaustive search for the right parameters. Cho et al. (2020) developed a Bayesian hyperparameter optimization for big data, and Tharwat and Hassanien (2019) used quantum-behaved particle swarm optimization, an evolutionary algorithm, to optimize the hyperparameters for deep neural networks. Random search, gradient-based, population-based and early stopping-based are other common examples of parameter optimizations algorithms. Similar to feature selection, we note that it is recommended to use nested resampling methods in combination with hyperparameter optimization to avoid overfitting in the ML model (Bischl et al. 2012).

### Reproducibility in stochastic models

Many ML algorithms are stochastic with random initialization, which is why different seeds can lead to different results. A fixed seed can be chosen to guarantee reproducible results from stochastic algorithms. This allows a scientist to later repeat the exact same random number generation involved. However, when assessing the algorithm’s performance and to avoid that a result reflects a local optimum instead of the true performance, it is recommended to systematically test multiple seeds to verify whether all models perform similarly regardless of the stochastic choice of a seed.

## Challenges in interpretability and evaluation

After running the ML methods and ensuring reproducibility when using stochastic ML models, it is important to be able to interpret and evaluate the results. Visualizing the results, for example using dimensionality reduction techniques, is an important aspect in interpretation, whereas evaluation metrics and benchmarking are vital for evaluation. The interpretability of an ML method is sometimes not evident. While some attempts have been made to improve the interpretability of ML methods, this is often negatively correlated with accuracy (Ahmad et al. 2018). Alternatively, some researchers argue that AI should not be interpretable as long as it is used under complete human supervision, and from the patients’ perspective, the efficacy is much more important (Jia et al. 2020). However, interpretable models are favored, and from a clinical point of view, having an idea of what a certain decision is based on, will improve the adoption by the medical community.

### Evaluation metrics

Evaluation metrics are essential when performing translational ML, since they offer a way to convey how well the trained model performed. Again here, the best evaluation metrics to use will depend on the data, the problem formulation and the ML models used.

Many unsupervised clustering metrics exist; however, these metrics are often complementary, as they do not tend to agree among themselves or with supervised evaluation criteria (Wiwie et al. 2015; Duò et al. 2020). An example of an evaluation metric is the Davies–Bouldin index. This evaluation metric takes into consideration the ratio of the between-cluster and within-cluster distances (Davies and Bouldin 1979). The silhouette index, another metric, measures how similar a sample is to its own cluster compared to other clusters (Rousseeuw 1987). A disadvantage of the silhouette index is that it is more computationally intensive. Dimensionality reduction is often scored subjectively. However, some metrics are available, such as the co-ranking matrix, which visualizes all neighbors of a point in high versus low dimensions. More quantitative metrics are trustworthiness and continuity (Kaski et al. 2003), the Local Continuity Meta Criterion (LCMC) (Chen and Buja 2009) and the mean relative rank errors (Lee and Verleysen 2009). These quantify how well the structure of original data is preserved in the lower dimensional embedding. While the LCMC is computed from what happens in the k-ary neighborhood only, the other metrics require the full co-ranking matrix.

An example of evaluation metrics in supervised learning is the area under the receiver-operating characteristic (AUROC) curve which measures the performance of an ML model by calculating the true-positive rate (TPR) or sensitivity and the false-positive rate (FPR) at different decision thresholds. The ROC curve gives an overview of all these possible decision thresholds, and so, one can balance the TPR and FPR oneself (Davis and Goadrich 2006). Typically, the balance between type I errors, or the false positives, and type II errors, or the false negatives, is application-dependent and should always be adapted to the specific clinical use case. If the ML algorithm is, for example, used to detect a subset of high-risk patients for further diagnostic testing, avoiding false negatives will be more important. The F1-score is the harmonic mean of precision and recall, which are both calculated with the number of true positives, true negatives, and false negatives. Challenges arise with both scores when they are applied on imbalanced datasets, meaning that there is an unequal class distribution. In this situation, balanced accuracy and the area under the precision and recall (AUPR) curve are more informative.

As each evaluation metric has both advantages and disadvantages and highlights different parts of a model's performance, there is no ultimate evaluation metric suited for every situation. In most cases, it is recommended to look at multiple metrics before making conclusions (Handelman et al. 2018).

### Benchmarking

Since there are numerous ML methods available, recent studies have focused on benchmarks (Weber and Robinson 2016; Saelens et al. 2019; Liu et al. 2019; Chazarra-Gil et al. 2021). These benchmarks often rely on a combination of synthetic and public datasets. While synthetic data can have important advantages to explore specific questions, as all of its properties are tunable, it remains difficult to ensure that it completely captures the intricacies of real data, especially in disease settings. Public datasets are more favorable and are available on online repositories. FlowRepository, CytoBank and ImmPort are databases that provide cytometry data, whereas the Single-Cell Expression Atlas and the Gene Expression Omnibus have single-cell sequencing data. However, it is noted in Volkmann et al. (2019) that only a few large clinical datasets acquired by omics technologies have been made publicly available, and the situation in the single-cell field is similar. We highly recommend data sharing as a way to improve translational ML research.

### Replicability, stability and confidence estimates

Some desirable properties of ML and statistical models, in general, are replicability and stability. We define replicability as the ability to replicate the performance of the ML model in a different cohort of patients, which will be limited if a model is overfitted on a specific dataset. In ML, this is also known as the external validation of the model, and it can be particularly challenging in a clinical setting.

The stability of an ML model refers to how much small changes in the training set impact the model (Evgeniou et al. 2004). Unstable models show much variation as a function of the specific training samples. A popular method to assess model stability is validation using bootstrap resampling, which uses a set of randomly drawn samples with replacement. In the case where leaving out a few samples completely changes the performance of the model, probably outliers are driving the model, making it unstable and less trustworthy.

Finally, while often only the predictions of ML algorithms are evaluated, some supervised models can also estimate the uncertainty of their predictions themselves. If these confidence estimates are well calibrated, it can be a valuable resource to guide clinicians in how much trust they can put in a specific prediction, rather than the whole model at once, and whether any further tests would be necessary in that specific instance or not. When evaluating the uncertainty estimates, different types of uncertainty can be distinguished, such as reducible uncertainty (e.g., due to limited sample size) and irreducible uncertainty (e.g., due to stochastic dependencies between instances and outcomes) (Hüllermeier and Waegeman 2021).

### Sensitivity analysis

Another option to assess the robustness of the ML algorithm is to change the input or feature space, also known as a sensitivity analysis. This is a simple, yet very useful technique to examine the impact of each feature on the ML model and is, for example, used to evaluate neural networks in image segmentation (Ankenbrand et al. 2021). One or more features are transformed, e.g., uniform resampling, permutation or other transformations, and the outcome of the non-transformed versus the transformed feature space is compared. If no substantial differences occur, the specific feature has little impact on the outcome of the ML algorithm. This can help to find which features contribute to the ML model and give more insight into the black box. Of particular interest are transformations that reflect a violation of the underlying assumptions of the algorithm or that induce specific patterns of missingness. Only if equivalent results are obtained, the algorithm can be applied in these additional scenarios.

### Biomarker discovery

ML models can be hard to interpret, so besides adopting the ML model itself in the clinic, it is also possible to apply ML to identify novel biomarkers which are measurable indicators of the relevant biological condition. These are easier to implement in the clinic as they can be less expensive, easier to interpret and can be less time-consuming. Nevertheless, biomarkers must be highly sensitive, highly specific, easily detectable by clinical assays and cost-effective (Gupta et al. 2014). In biomarker discovery, feature extraction techniques can extract potential biomarkers from the cleaned data, and feature selection techniques can be applied to these biomarkers to rank them according to importance. Xie et al. (2021) extracted 61 metabolites levels from metabolomics data and afterward, used a fast correlation-based filter for feature selection. The top five features could potentially be used to detect early lung cancer. A study by Mamoshina et al. (2018) used neural networks on publicly available transcriptomic data profiles to identify tissue-specific biomarkers for aging and demonstrated that these biomarkers could be used to identify new molecular anti-aging therapies. Naturally, these biomarkers also need to be validated later on in the clinic on new samples before they can be adopted. To use these biomarkers routinely in the clinic, it might be necessary to develop a new assay, for example, an RT-PCR test for genes selected based on a single-cell RNA-sequencing experiment.

## Challenges in adopting ML in the clinic

### External validation and reporting

Before adopting the ML model in the clinic, it first needs to be externally validated. The model needs to be tested on independent and larger cohorts, and on the other hand, it needs validation on a cohort from an independent institute. The samples and the associated data of this latter cohort are collected by an entirely new set of staff. An example that shows the importance of external validation can be found in Zech et al. (2018) where the authors wanted to diagnose pneumonia based on chest radiographs with the help of a CNN. The CNN predicted pneumonia with significantly lower performance when using data from another hospital, while it could accurately predict the hospital where the data came from (Zech et al. 2018).

Multiple guidelines and checklists exist to report an ML model, not only for single-cell data but also for AI in omics data (Collins et al. 2021). Wynants et al. (2020) validated ML prediction models concerning COVID-19 using a CHecklist for critical Appraisal and data extraction for systematic Reviews of prediction Modeling Studies (CHARMS, Moons et al. 2014) and the Prediction model Risk Of Bias ASsessment Tool (PROBAST, Moons et al. 2019). This review also mentioned that the Transparent Reporting of a multivariable prediction model for Individual Prognosis Or Diagnosis guidelines should be followed when reporting and validating prediction models to increase interpretability, reproducibility and reportability (TRIPOD, Moons et al. 2015; Wynants et al. 2020). PROBAST and TRIPOD are currently also being expanded to include AI studies (Collins et al. 2021). Heil et al. (2021), Walsh et al. (2021) and Matschinske et al. (2021) also propose recommendations and reporting methods for machine learning in life sciences.

It is also advised to compare the ML model performance with established clinical scores or existing ML models. Duetz et al. (2021), for example, validated their model in an external cohort and also noticed that their ML model outperformed the expert-analyzed flow cytometry score to classify myelodysplastic syndrome (MDS) from non-MDS.

### Unconscious bias

When training ML models, spurious associations can be picked up due to confounders, such as age, gender, race or the place where the samples were acquired, etc. that influence the association between biomedical features and the outcome of interest. If a causal relationship is attributed to these spurious associations or if these associations are not properly corrected for confounders in the model, they can induce bias when building the model and applying it in a clinical setting (Gianfrancesco et al. 2018).

To avoid bias, it is therefore crucial to measure and register all potential confounders. Spurious associations can even be induced by unmeasured confounders, resulting in unconscious bias. A study by Obermeyer et al. (2019) revealed racial bias, because an ML model predicted health care costs instead of the actual illness. In addition, the demographic space used to train the ML algorithm should be representative of the demographic space which will be present in the clinic to avoid a biased performance of the model. Buolamwini and Gebru (2018) found, for example, that dark-skinned women are more likely to be misclassified by facial analysis software due to an imbalance in the training dataset.

Good practice would be to assess all patient variables before starting the experiment to factor them out in the downstream analysis. However, assessing everything, for example with correlation plots, is practically impossible. One way to evaluate bias in ML is the use of counterfactuals, where artificial observations are created for a set of patients. Given that a potential confounder is changed, for example race, all other features are changed accordingly based on a probabilistic model. If the prediction of the ML model does not change, the confounder does not induce bias (Pfohl et al. 2019). While there also have been studies trying to debias ML models, these only had limited successes, and preventing the bias in the first place is strongly recommended (Eid et al. 2021).

### Intellectual property

Concerning intellectual property (IP) and AI, the World Intellectual Property Organization (WIPO) differentiates between AI-assisted versus AI-generated inventions. AI-assisted inventions are defined as tools generated by humans, while AI-generated inventions are created by AI. Whereas AI-assisted inventions follow the regular IP regulations, issues arise with AI-generated inventions, since it is difficult to state who the owner is; a human, the AI or a joint ownership (WIPO secretariat 2021). Current ML approaches are mostly AI-assisted and therefore are covered by the regular IP regulations.

### Trustworthy and explainable AI

Even if the ML model is proven to be valuable and can generalize to unseen samples or other cohorts, clinicians might still favor slightly worse, but explainable and interpretable models over “black box” models. Lack of trust in these complicated models is at the root of this preference. A recent study by Cheung et al. (2021) surveyed the current trends in computational flow cytometry and found out that indeed the primary reason that automated analysis was not used is lack of trust but also lack of understanding and resources. The pursuit of trustworthiness in ML is currently a big topic, and this also applies in translational ML. Consequently, we propose steering towards more interpretable and explainable AI models (Quinn et al. 2021). Alternatively, trust can be gained by evaluating whether some of the features selected by the model are already known clinical parameters or can be easily related to such parameters. In Garzorz-Stark et al. (2016), for example, a logistic regression model was built to distinguish psoriasis and eczema using gene expression of two important genes which resulted in a high sensitivity after cross-validation.

### FDA and EMA approval

Before an ML algorithm can be implemented as a medical decision tool in the clinic, it needs to be approved by the authorities such as the FDA or the EMA. Results of translational ML need to be robust and generalize well to the intended population. A review paper by Wu et al. (2021) collected all FDA-approved medical AI devices that were approved between January 2015 and December 2020. They noticed that 126 of the 130 evaluations only underwent retrospective studies and that the number of evaluation sites is often not reported which can lead to restricted diversity in geography (Kaushal et al. 2020; Wu et al. 2021). An example of FDA-approved software as a medical device is IDx-DR which uses a CNN to autonomously detect diabetic retinopathy (Abràmoff et al. 2016; Savoy 2020). Afterward, post-market surveillance must be established by sharing the results of the clinic with the bioinformatician to anticipate unintended outcomes and biases that were not detected earlier (Ferryman 2020).

## Conclusion

Even though ML is already quite common in translational research, there is still room for improvement, both on the wet lab and the computational side. In this review, we address the challenges that arise in translational machine learning and anticipate that the way forward to more successful clinical applications is the construction of large consortia that are able to generate sizable patient cohorts in a standardized fashion. We refer to several examples of translational single-cell studies throughout the work, extended with some more general imaging and omics applications in those cases where single-cell technologies are still in the process of being adopted in the clinic. As these technologies are ever-evolving, we also expect new techniques, such as spatial transcriptomics at single-cell resolution or combinations of techniques (multi-omics) to be adopted in clinical settings in the near future. Additionally, we also expect other ML techniques, such as semi-supervised learning and reinforcement learning, soon to be translationally applied to single-cell datasets. We highly recommend starting with a multi-disciplinary team consisting of clinicians, bioinformaticians and biostatisticians before planning the experiment to get acquainted with which ML techniques apply to a specific translational research question and to optimize the experimental design and sample size. Other valuable aspects to keep in mind before starting the experiment are privacy, ethics, regulations and legislation. Data can only be used from patients who completed an informed consent form which states that they agree that their data will be used for research and might be published in a pseudonymized version. As previously mentioned, the pseudonymization of data is not an easy-to-solve problem, and it opens up the debate between patient privacy versus open science, where sharing anonymized patient data is stimulated to allow further research and meta-analysis.

Many ML models are available and choosing the right one is not a trivial question. It depends on the hypothesis, the amount of data, how the data are balanced, if there is clinical metadata available, the need for interpretability of the results, privacy concerns, etc. New ML techniques are also coming out faster than ever, so keeping up with the literature as well as checking recent benchmarks will provide more guidance to select the right ML technique. On top of that, it is recommended to test multiple ML models. Once the models are selected, the computational pipeline can be assessed with resampling methods, such as cross-validation. These help to verify that the model is not overfitting and help to assess generalizability. It is important to include feature selection and hyperparameter tuning inside the (nested) resampling procedure. Quality control is an important step in translational ML as it identifies noise and bad quality data. Once the ML models are set up, they need to be evaluated and interpreted. This requires generalizable evaluation metrics, sensitivity analysis and a strong analysis concerning replicability, stability and reliability. Future research is still necessary on how to balance the trade-off between complex models and interpretability while avoiding unconscious bias.

After building the model, it is of utmost importance that it not only gets validated on an internal dataset but also on external ones to ensure that models perform well in a wide variety of settings. Trustworthiness can also be increased by linking the outcomes of the ML model to parameters known by clinicians when possible, even though parameter linking is less evident in multivariate models. The next step is to get the ML model approved by the authorities, such as the FDA and EMA. This, at last, includes post-market surveillance, where feedback from the clinic can be used to improve the model. Overall, we would argue that single-cell data of large cohorts with appropriate privacy measures and explored by several ML models will lead to relevant clinical tools, allowing more accurate diagnosis and prognosis. We expect many more to be approved by authorities and health care instances in the coming years, ultimately benefiting the patients.

## Data Availability

Not applicable.
